# Cost-effectiveness of a programme of screening and brief interventions for alcohol in primary care in Italy

**DOI:** 10.1186/1471-2296-15-26

**Published:** 2014-02-06

**Authors:** Colin Angus, Emanuele Scafato, Silvia Ghirini, Aleksandra Torbica, Francesca Ferre, Pierluigi Struzzo, Robin Purshouse, Alan Brennan

**Affiliations:** 1 School of Health & Related Research (ScHARR), University of Sheffield, Sheffield, UK; 2 Istituto Superiore di Sanità, Viale Regina Elena, Rome, Italy; 3 Centre for Research on Health and Social Care Management (CERGAS) Bocconi University, Via Roentgen, Milan, Italy; 4 Region Friuli Venezia Giulia, Regional Centre for the Training in Primary Care, Monfalcone, Italy; 5 Department of Automatic Control & Systems Engineering, University of Sheffield, Sheffield, UK

**Keywords:** Public health interventions, Alcohol, Primary care, Cost-effectiveness

## Abstract

**Background:**

As alcohol-related health problems continue to rise, the attention of policy-makers is increasingly turning to Screening and Brief Intervention (SBI) programmes. The effectiveness of such programmes in primary healthcare is well evidenced, but very few cost-effectiveness analyses have been conducted and none which specifically consider the Italian context.

**Methods:**

The Sheffield Alcohol Policy Model has been used to model the cost-effectiveness of government pricing and public health policies in several countries including England. This study adapts the model using Italian data to evaluate a programme of screening and brief interventions in Italy. Results are reported as Incremental Cost-Effectiveness Ratios (ICERs) of SBI programmes versus a ‘do-nothing’ scenario.

**Results:**

Model results show such programmes to be highly cost-effective, with estimated ICERs of €550/Quality Adjusted Life Year (QALY) gained for a programme of SBI at next GP registration and €590/QALY for SBI at next GP consultation. A range of sensitivity analyses suggest these results are robust under all but the most pessimistic assumptions.

**Conclusions:**

This study provides strong support for the promotion of a policy of screening and brief interventions throughout Italy, although policy makers should be aware of the resource implications of different implementation options.

## Background

The negative health and social impacts of excessive alcohol consumption are well-documented and place a heavy cost-burden on society [1]. Drinking has been identified as a contributory factor in a wide range of health conditions [2] and as a high priority cause of chronic disease in Italy [3]. As a result, the attention of policymakers is increasingly turning to screening and brief intervention (SBI) programmes. The recent World Health Organisation (WHO) European Action Plan to Reduce the Harmful Use of Alcohol [4] recommends such programmes as a key strategy for health services throughout the continent and the use of SBIs in primary care is currently recommended in Italian national guidelines [5]. However, whilst the effectiveness of SBI in reducing alcohol consumption is well-evidenced [6-8], recent systematic reviews of cost-effectiveness literature [9,10] have identified few studies into the cost-savings resulting from SBI programmes, none of which which focus on the Italian context. This lack of evidence may be acting as a significant barrier to the wider implementation of SBIs by the regional bodies in Italy responsible for public health.

This study forms part of the European Commission funded Optimising Delivery of Healthcare Interventions (ODHIN) project, which uses SBI in primary healthcare as a case study to investigate how primary healthcare professionals can be encouraged to implement practices which clinical research shows to be effective. Recent modelling work has shown SBI programmes in primary healthcare to be highly cost-effective in England [11] and this study adapts this work using Italian data to estimate the cost-effectiveness of SBI programmes in primary healthcare in Italy.

Our analysis evaluates two alternative SBI policies. We considered the uncertainty around the existing evidence by examining a range of sensitivity analyses, as well as investigating the impact of using alternative screening tools. Our results are then compared with those of recent cost-effectiveness studies in other countries.

## Methods

### Model structure

**Figure 1:**
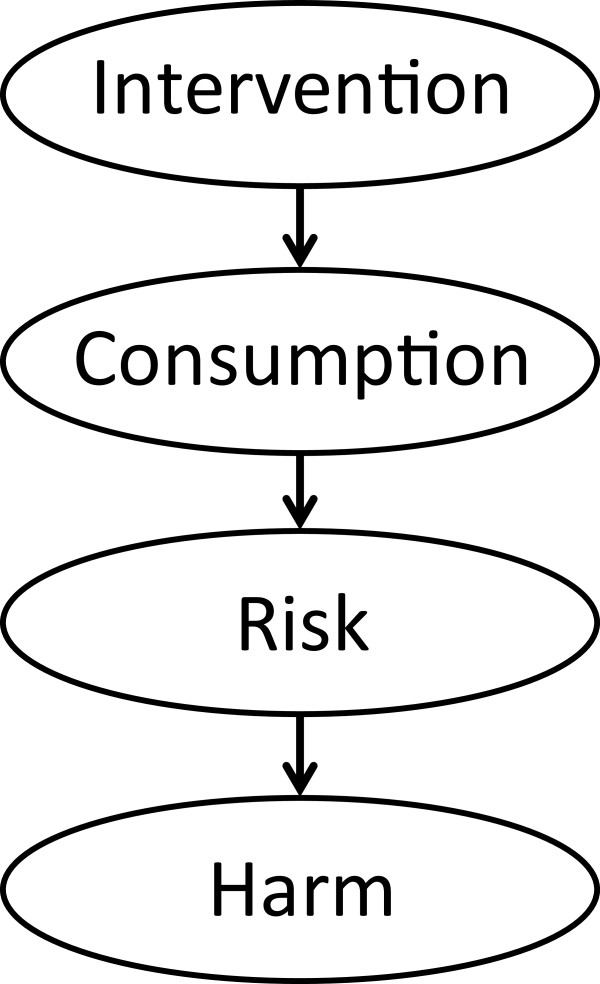
High-level conceptual framework for the model.

We adapted an existing alcohol policy appraisal model - the *Sheffield Alcohol Policy Model* - which has been used to evaluate pricing policies in England and Scotland [12-14] and screening and brief intervention policies in England [15]. This model takes existing alcohol consumption in the population and estimates how this changes over time as the result of an intervention. These changes are then used to estimate the impact on population health. The high-level conceptual framework of the model is shown in Figure 1.

We modelled a 10 year programme of screening and brief interventions in primary healthcare with a 30 year time horizon to allow the full impact on health outcomes to be considered. The model utilises an adapted form of Gunning-Scheper’s method of potential impact fractions and alcohol-attributable fractions (AAFs) [16] to estimate the change in mortality and morbidity rates resulting from changes in consumption (see pages 25–36 of Purshouse et al. [15] for full details). These rates are used to adjust the population life tables and obtain revised levels of mortality and morbidity for a range of health conditions, which can then be compared to a no intervention scenario. Health outcomes are calculated on a population subgroup level, with subgroups defined by age (16–17, 18–24, 25–34, 35–44, 45–54, 55–64, 75+), sex and mean alcohol consumption at baseline (measured in grams of pure ethanol/week).

Screening is modelled by constructing a screening arrival profile which represents the proportion of each age and gender subgroup being screened in each year of the 10 year programme. Each individual in the population, represented in the model by an individual survey respondent, can be screened no more than once and it is assumed that all individuals are screened at the first opportunity. The actual samples in each subgroup selected for screening in each year are chosen randomly, accounting for sample weights. A more in-depth explanation of the full modelling methodology can be found elsewhere [11,15].

The perspective for this analysis is that of the national healthcare system, including cost savings to the Italian National Health Service (INHS) as a result of reduced morbidity and improvements in health-related quality of life for drinkers as well as the direct costs of illness and the opportunity costs of delivering the SBIs in primary care. All costs and health outcomes are discounted at a rate of 3%, with values of 0% and 5% tested as sensitivity analyses in line with current Italian guidance [17], and all costs are presented in 2008 prices.

### Italian model parameters

#### Consumption data

We obtained consumption data for the Italian population from the Aspects of Daily Life survey 2008 [18,19], conducted by the Italian national statistics institute (ISTAT). This nationally representative survey (N = 48,861) records demographic data on each respondent as well as asking a series of quantity-frequency questions regarding their usual alcohol consumption. These responses were converted to a mean weekly consumption in grams of alcohol. The survey also asks respondents how many times in the preceding year they have drunk 6 or more glasses of alcoholic beverage (1 glass = 12 grams of pure alcohol). We used this as a measure of the risk of harm for health conditions associated with acute, rather than chronic, alcohol consumption.

#### Mortality and morbidity data

We modelled 42 alcohol-attributable health conditions based on the work of the WHO on the global burden of disease due to alcohol [20,21] (see Table 1). Absolute mortality data from 2008 for the 42 health conditions was obtained from ISTAT for each age and gender subgroup in the model. Morbidity data was derived from the Italian database of hospital admissions for 2008, which contained a total of 1,559,310 admissions for the 42 alcohol-related health conditions. In order to account for repeat admissions in the same year for the same individual, the absolute number of admissions was divided by the adjustment coefficients shown in Table 1. These coefficients were calculated from hospital admissions data for the Netherlands as no Italian data was available and were obtained from the Dutch Hospital Data Foundation (DHD) [22]. UK coefficients were used in previous versions of the Sheffield Model [15], however these were estimated indirectly from several data sources, whereas the DHD data allowed them to be calculated directly. The coefficients represent the mean number of admissions in a year for an individual with each health condition and full details on their derivation are contained within the Additional file 1. Hospital admissions data for Italy was not available for any of the partially-attributable acute conditions, so these were estimated from UK morbidity data using the relationship between Italian and UK mortality rates for each relevant condition.

**Table 1:** Alcohol-attributable health conditions included in the model

**Health condition**	**ICD-10 code(s)**	**Total mortalities (2008)**	**Hospital admissions (2008)**	**Adjustment coefficient**	**Estimated morbidity (2008)**
	**Wholly alcohol-attributable chronic conditions**				
Alcohol-induced pseudo-Cushing's syndrome	E24.4	0	774	1.17	661
Degeneration	G31.2	29	284	1.10	257
Alcoholic polyneuropathy	G62.1	12	444	1.14	390
Alcoholic myopathy	G72.1	0	99	1.00	99
Alcoholic cardiomyopathy	I42.6	15	99	1.26	79
Alcoholic gastritis	K29.2	1	104	1.09	96
Alcoholic liver disease	K70	10	3283	1.51	2179
Chronic pancreatitis	K86.0	7	17901	1.47	12180
	**Wholly alcohol-attributable acute conditions**				
Excessive blood level of alcohol	R78.0	0	16	1.00	16
Mental and behavioural disorders due to use of alcohol	F10	236	13930	1.14	12242
Ethanol poisoning	T51.0	2	112	1.11	101
Methanol poisoning	T51.1	0	3	1.00	3
Toxic effect of alcohol, unspecified	T51.9	8	16	1.22	13
Accidental poisoning by exposure to alcohol	X45	0	0	1.03	0
	**Partially alcohol-attributable chronic conditions**				
Malignant neoplasm of lip, oral cavity and pharynx	C00-C14	2742	10467	1.59	6580
Malignant neoplasm of oesophagus	C15	1773	4340	2.19	1982
Malignant neoplasm of colon and rectum	C18-21	18471	68903	2.14	32157
Malignant neoplasm of liver and intrahepatic bile ducts	C22	9429	30738	1.59	19322
Malignant neoplasm of larynx	C32	1707	9046	1.47	6173
Malignant neoplasm of breast	C50	12298	69691	2.35	29605
Diabetes mellitus (typeII)	E10-E14	20170	110599	1.31	84107
Epilepsy and status epilepticus	G40-G41	659	31738	1.16	27276
Hypertensive diseases	I10-I15	21315	117558	1.19	99091
Ischaemic heart disease	I20-I25	75044	350551	1.19	294472
Cardiac arrhythmias	I47-I49	7806	168777	1.27	132619
Haemorrhagic stroke	I60-I62	10611	34635	1.07	32320
Ischaemic stroke	I63	1974	260020	1.07	243881
Oesophageal varices	I85	67	2300	1.50	1534
Unspecified liver disease	K74	6986	60244	1.32	45709
Cholelithiasis	K80	629	137456	1.16	118453
Acute and chronic pancreatitis	K85, K86.1	1052	10711	1.10	9708
Psoriasis	L40 excl. L40.5	23	17017	5.74	2966
Spontaneous abortion	O03	0	32258	1.05	30616
	**Partially alcohol-attributable acute conditions**				
Motor vehicle accidents	V0-V04, V06, V09-V80, V87, V89, V99	4712	N/A	1.05	40280
Fall injuries	W00-W19	2910	N/A	1.05	278333
Drowning	W65-W74	312	N/A	1.00	212
Fire injuries	X00-X09	212	N/A	1.12	2080
Accidental poisoning by exposure to noxious substances	X40-X49	468	N/A	1.03	3723
Other unintentional Injuries	V05, V07, V08, V81-V86, V88, V90-V98, W20-W64, W75-W99, X10-X39, X50-X59, Y40, Y86, Y88, Y89	10392	N/A	1.06	26458
Intentional self-harm	X60-X84, Y87.0	3794	N/A	1.15	35440
Assault	X85-Y09, Y87.1	427	N/A	1.04	42681
Other intentional injuries	Y35	0	N/A	1.10	505

#### Consumption-risk relationships

Risk functions relating alcohol consumption (either mean consumption level or frequency of acute drinking) to risk are required to implement the potential impact fraction method. Following a recent review by the Sheffield Alcohol Research Group, updated risk functions were identified for malignant neoplasm of the lip, oral cavity and pharynx [23], malignant neoplasm of the liver and intrahepatic bile ducts [24], malignant neoplasm of the larynx [25], malignant neoplasm of the breast [26], epilepsy [27] and cardiac arrhythmias [28]. For all other partially-attributable chronic conditions the same risk functions as have been implemented previously [29] were used. For partially attributable acute conditions (e.g. falls and assaults) we used Italy-specific alcohol-attributable fractions [19] where available, or otherwise those from England [30], and assumed a linear relationship between frequency of drinking >6 glasses and risk to calculate risk functions for each age-gender subgroup. For wholly-attributable partial and chronic conditions, the risk functions were calibrated to the actual absolute levels of morbidity and mortality in the population, assuming a linear relationship between alcohol consumption/binge frequency and risk. Further details of the calibration methodology and risk functions used are included in the Additional file 1.

#### Healthcare costs of alcohol-attributable health conditions

The model incorporates all healthcare costs to the INHS for each health condition, including inpatient, outpatient and accident and emergency visits, ambulance costs, GP consultations, nurse visits and other costs. For each cost category identified, we aimed to collect context-specific costs in line with the study perspective. In Italy, hospitals are funded through reimbursement tariffs that vary across 21 regions and between different types of providers. The reimbursement tariffs are estimated on the basis of full cost of hospitalisation and are inclusive of all inpatient services in addition to emergency visits if these led to hospitalisation [31]. Since we aimed to obtain nationally representative estimates, we assigned hospital costs to each admission in the database based on the nationally defined reimbursement tariff which applies when a patient is treated outside their region of residence. Out of 42 identified alcohol-attributable conditions, Italian data were available for 33. Mean costs were calculated for each condition by gender and tested for significant differences between sexes using student t-tests in Stata 11 [32]. Costs were significantly different (p < 0.01 in all cases) for 11 of the conditions for which data was available. Where there was no significant difference (p > 0.23 in all cases) the overall population mean was used. For conditions where no cost data was available in Italy, English costs [15] were adjusted by the mean ratio between Italian and English costs for all other conditions. This assumption was tested with a sensitivity analysis assuming, conservatively, that these costs were 25% lower than the baseline estimates. Costs not covered within the hospital reimbursement tariffs (e.g. ambulance and GP costs) were estimated assuming the ratio of hospital admission to other costs for each condition was the same as in England.

#### Costs of screening and brief intervention

The costs associated with implementing a screening and brief intervention programme were separated into the cost of briefing materials provided to the patient and the cost of the GP’s time. The former were taken from a UK study by Lock et al. [33], converted into euros using OECD purchasing power parities [34] and inflated to 2008 prices using the consumer prices index for Italy [35]. To obtain an estimate of the GP’s time, we first estimated the annual salaries of GPs of different levels of seniority, using data from the Friuli-Venezia-Giulia (FVG) region of Italy. We took an average of these, weighted by the proportion of GPs at each level in the province of Udine (in the FVG region) to give us an estimate for the mean annual salary of a GP of €79900 before tax. Italian GPs spend an average of 15 hours contact time in surgery with patients [36] and an estimated 12.5 hours on home visits, giving us an average direct cost per minute to the INHS of €0.87 for this contact time (after adjusting to 2008 prices). As no data exists on the costs of overheads and other related costs (such as ongoing training) these were estimated relatively from UK figures [37], giving a total cost per minute to the INHS of €1.07. Owing to the uncertainty around this figure, an alternative estimate of €1.58, derived using the absolute UK costs, was used as a sensitivity analysis.

#### Screening coverage

Two alternative intervention settings were examined: the screening of patients when they next registered with a new GP and screening when they have their next standard GP consultation. Data on between-GP migration by patient age and gender was obtained for the Friuli-Venezia-Giulia region for a 10 year period from 2000–2009 (personal communication from Roberto Maffetone at INSIEL) together with regional population demographics for the same period from ISTAT. These were used to derive a gender and age-group specific ‘arrival profile’, after adjusting for long-term trends in migration, giving the probability of being screened in each year of a 10 year screening programme, assuming that the probability of registering with a new GP was independent from year to year. Data on the mean frequency of GP consultations by age and gender was obtained for both Italy [38] and England [39] and used to estimate the proportion of patients in each subgroup who would visit their GP in each year of the programme. Full details of the methodology used in this estimation are given in an additional file [Additional file 1].

In line with current Italian guidelines, screening is modelled using the AUDIT-C questionnaire with a threshold of 4 for women and 5 for men [40] with a brief intervention lasting 10 minutes for all patients who screen positive. The probability of a patient screening positively, given their alcohol consumption, was estimated using screening models derived from the UK Psychiatric Morbidity Surveys from 2000 and 2007. Evidence on the relationship between duration of intervention and effectiveness is inconclusive [7] and therefore the mean effect estimate of a 12.3% reduction in consumption after a 24.9 minute intervention, taken from the Cochrane review of Kaner et al. [6], is applied to all patients who receive the intervention. This reduction is assumed to rebound linearly towards the patient’s baseline consumption over a period of 7 years in line with the findings of Fleming et al. [41].

In order to investigate the impact of uncertainty around the hospital costs estimated indirectly from UK costs, the cost of GP’s time, the duration and magnitude of effect of the intervention on patients’ drinking and the relationship between these and the duration of the brief intervention itself, we conducted a number of sensitivity analyses using more pessimistic assumptions for these parameters. A number of alternative scenarios were also examined to explore the estimated impact of using different screening tools, such as the full AUDIT questionnaire, FAST, which has been recommended for use in primary care [42], or combinations of several tools (e.g. full AUDIT with an AUDIT-C pre-screen).

## Results

### Population coverage

**Figure 2:**
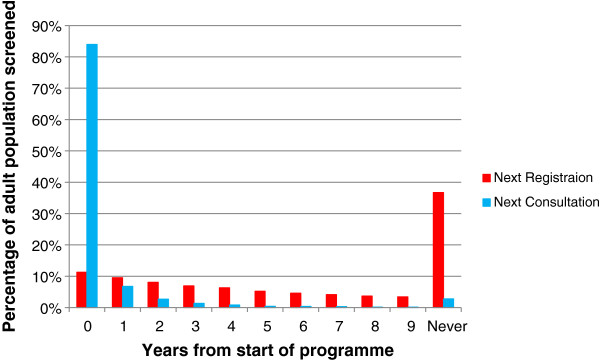
Population coverage of modelled screening programmes.

The population coverage for a programme of screening at next GP registration is estimated to be 63% of the total adult population, leading to 32% of people receiving a brief intervention during the 10 years of the programme. Coverage is spread relatively evenly across the 10 years, peaking in year 1 with 11% of the population being screened. A programme of screening at next consultation is estimated to capture 97% of the population over 10 years, with 49% of adults receiving an intervention as a result; however this is heavily loaded towards the start of the programme, with 84%% of people being screened in the first year. Figure 2 shows these coverage profiles over the lifetime of the programme.

### Screening at next GP registration

Over the course of 30 years, a programme of screening at next GP registration is estimated to result in 7200 fewer alcohol-attributable deaths, predominantly amongst men (66%) and from chronic (68%), rather than acute causes. The total number of hospitalisations saved by the programme is estimated to be 91700, also largely amongst men (72%) and for chronic conditions (67%). Table 2 gives a detailed breakdown of the estimated impact on alcohol-related morbidity in the fifth year of the programme.

**Table 2:** Estimated reductions in morbidity (absolute and relative to baseline) in the 5th year of a programme of SBI for patients being screened at their next GP registration

	**16-24 years**				**25-44 years**				**45-64 years**				**65 years or older**				**Total**					
	**M**		**F**		**M**		**F**		**M**		**F**		**M**		**F**		**M**		**F**		**All**	
**Condition**	**N**	**%**	**N**	**%**	**N**	**%**	**N**	**%**	**N**	**%**	**N**	**%**	**N**	**%**	**N**	**%**	**N**	**%**	**N**	**%**	**N**	**%**
Alcoholic poisoning	0	-1.7%	0	-3.1%	-1	-3.1%	0	-4.9%	-1	-4.0%	-2	-12.2%	-1	-6.5%	0	-2.4%	-2	-3.5%	-2	-6.4%	-4	-4.6%
Alcoholic disorders	-12	-2.2%	-9	-2.8%	-204	-3.3%	-93	-4.0%	-259	-3.3%	-182	-5.5%	-132	-2.9%	-104	-2.6%	-608	-3.2%	-388	-3.9%	-996	-3.4%
Assault	-59	-0.4%	-16	-1.0%	-199	-1.1%	-52	-2.0%	-73	-1.7%	-13	-1.9%	-17	-2.3%	-4	-0.8%	-348	-0.9%	-84	-1.6%	-432	-1.0%
Road traffic accidents	-92	-1.2%	-10	-0.5%	-151	-1.2%	-16	-0.5%	-52	-0.9%	-8	-0.4%	-23	-0.8%	-9	-0.4%	-318	-1.1%	-44	-0.4%	-362	-0.9%
Epilepsy	-30	-1.4%	-2	-0.1%	-46	-1.5%	-3	-0.1%	-29	-0.9%	-6	-0.3%	-29	-0.8%	-5	-0.1%	-134	-1.1%	-16	-0.1%	-150	-0.6%
Other accidents	-37	-0.3%	-12	-0.3%	-217	-0.8%	-74	-0.7%	-232	-1.0%	-129	-0.8%	-894	-1.3%%	-168	-0.1%	-1380	-1.1%	-382	-0.2%	-1762	-0.6%
Intentional self harm	-6	-0.2%	-18	-0.3%	-36	-0.5%	-64	-0.7%	-20	-0.7%	-25	-0.7%	-12	-1.0%	-5	-0.3%	-74	-0.5%	-112	-0.6%	-186	-0.5%
Diseases of the digestive system	-5	-0.5%	1	0.0%	-82	-0.6%	6	0.0%	-145	-0.5%	2	0.0%	-102	-0.3%	-27	-0.1%	-334	-0.4%	-19	0.0%	-353	-0.2%
Diseases of the circulatory system	-27	-1.3%	-1	-0.1%	-82	-0.4%	-11	-0.1%	-578	-0.4%	-218	-0.3%	-398	-0.1%	-211	-0.1%	-1085	-0.2%	-440	-0.1%	-1525	-0.2%
Neoplasms	0	-0.2%	0	-0.1%	-3	-0.3%	-5	-0.1%	-52	-0.3%	-14	-0.1%	-16	0.0%	-11	0.0%	-71	-0.1%	-30	0.0%	-101	-0.1%
Other	-1	-0.3%	0	0.0%	-6	-0.4%	-2	0.0%	-5	-0.2%	-2	-0.1%	-2	-0.2%	-1	-0.1%	-14	-0.3%	-4	0.0%	-18	0.0%
Diabetes	3	0.1%	0	0.0%	11	0.2%	1	0.0%	28	0.2%	2	0.0%	27	0.1%	3	0.0%	69	0.2%	7	0.0%	76	0.1%
Total	-266	-0.6%	-66	-0.3%	-1016	-0.8%	-312	-0.3%	-1418	-0.5%	-596	-0.4%	-1599	-0.3%	-540	-0.1%	-4299	-0.5%	-1514	-0.2%	-5813	-0.3%

Included ICD-10 codes: (1) T51.0, T51.1, T51.9, X45; (2) E24.4, R78.0, F10, G31.2, G62.1, G72.1, I42.6, K29.2, K70, K86.0; (3) X85-Y09, Y87.1; (4) V0-V04, V06, V09-V80, V87, V89, V99; (5) G40-G41; (6) W00-W19, W65-W74, X00-X49, V05, V07, V08, V81-V86, V88, V90-V98, W20-W64, W75-W99, X10-X39, X50-X59, Y40, Y86, Y88, Y89; (7) X60-X84, Y87.0; (8) I10-I15, I20-I25, I47-I49, I60-I63; (9) I85, K74, K80, K85, K86.1; (10) C00-C15, C18-22, C32, C50; (11) L40 excl. L40.5, O03, Y35 (12) E10-E14.

The cost of delivering SBIs over the 10 year programme is estimated to be €411 million. This is offset by a total reduction in hospital costs over 30 years of €370 million. The total gain in Quality-Adjusted Life Years (QALYs) is estimated to be 75200 giving an Incremental Cost-Effectiveness Ratio (ICER) of €550/QALY, suggesting that such a programme is close to being cost-neutral. As a large proportion of the health benefits are experienced by men (69% of total QALYs), delivering SBIs to men only is estimated to be cost-saving, although the estimated ICER for a female-only SBI programme of €3100/QALY is still well within the recommended Italian threshold of €25000-€40000/QALY [43].

### Screening at next GP consultation

**Figure 3:**
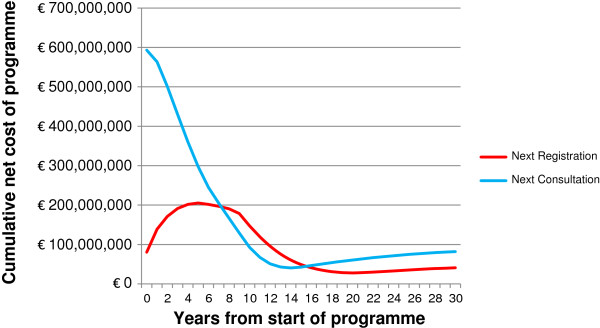
Cumulative net costs of modelled screening programmes (implementation costs and cost-savings to INHS).

As a programme of SBI at next GP consultation has a wider coverage, it is estimated to produce even greater improvements in public health, with 12400 fewer alcohol-attributable deaths and 153700 fewer hospital admissions over 30 years. The cost of delivery is also higher, at €687 million, although this is offset by cumulative healthcare savings of €605 million, making the programme around twice as expensive as screening at next registration. Health savings are estimated to be 139200 additional QALYs, giving an ICER of €590/QALY and suggesting there is little to choose between the two programmes in terms of cost-effectiveness. It should be noted that as the majority of SBIs take place in the first year of the programme, the bulk of the delivery costs are incurred up front, whilst the healthcare savings are accrued over a longer time frame. This is in contrast to screening at next registration, where the SBI costs are spread more evenly across the duration of the programme. Figure 3 shows the cumulative net costs of both programmes over time.

### Sensitivity analyses - discount rate

In order to investigate the sensitivity of model results around the baseline Italian discount rate of 3%, guidelines recommend alternative rates of 0% and 5% being tested. For a programme of screening at next registration a rate of 0% makes the programme cost-saving, whilst 5% increases the estimated ICER to €1200/QALY. For screening at next consultation similarly small effects are observed with 0% and 5% leading to estimates of €60 and €1100/QALY respectively.

### Sensitivity analyses - model assumptions

Whilst the assumptions in the base case scenarios are the best representation of the available evidence, a range of further analyses have been performed using more pessimistic assumptions around the size of effect and duration of effect of the intervention as well as the length of the intervention and the staff costs of the GPs delivering the SBIs. Results of these analyses are presented in Table 3, showing that even under the most pessimistic of assumptions is it likely that either programme would be considered cost-effective under current Italian guidelines [43].

**Table 3:** Impact of pessimistic alternative assumptions for SBI delivery costs and effectiveness estimates: ICERs versus a ‘do-nothing’ scenario

**Next registration**	**Base case**	**Lower hospital costs (-25% for estimated values)**	**Higher GP costs (€1.58/min)**	**Longer intervention (24.9 min)**	**Longer intervention, higher GP costs & cheaper hospital costs**
**Base case**	€ 550	€ 1000	€ 2000	€ 3900	€ 7400
**Less effective (5.9% reduction)**	€ 3600	€ 4100	€ 5900	€ 8800	€ 14000
**Shorter effect (3 year rebound)**	€ 7500	€ 8000	€ 10900	€ 15100	€ 22500
**Less effective & shorter effect (5.9% reduction & 3 year rebound)**	€ 14300	€ 14800	€ 19400	€ 26000	€ 37200
**Next consultation**	**Base case**	**Lower hospital costs (-25% for estimated values)**	**Higher GP costs (€1.58/min)**	**Longer intervention (24.9 min)**	**Longer intervention, higher GP costs & cheaper hospital costs**
**Base case**	€ 590	€ 950	€ 1900	€ 3600	€ 6700
**Less effective (5.9% reduction)**	€ 2500	€ 3700	€ 5400	€ 8000	€ 12600
**Shorter effect (3 year rebound)**	€ 5900	€ 7400	€ 10100	€ 13900	€ 20600
**Less effective & shorter effect (5.9% reduction & 3 year rebound)**	€ 11600	€ 13600	€ 17900	€ 23900	€ 33900

### Sensitivity analyses - alternative implementation options

Table 4 presents the results of a range of alternative model runs, showing the estimated impact of using alternative screening tools and thresholds. These results show that whilst the current recommended screening tool in Italy (AUDIT-C 5/4) is the most expensive to implement, it is also the most effective and produces the greatest net benefit for both screening at next registration and at next consultation. The results also illustrate the scale of potential net benefits from adopting a national SBI policy (estimated to be in excess of €1.3 bn at a willingness-to-pay threshold of €25000/QALY for any of the modelled scenarios).

**Table 4:** *Model results for alternative implementation scenarios, ordered by incremental net benefit*

	**Setting**	**Screening tool and threshold (M/F)**	**Delivery costs (€m)**	**INHS savings (€m)**	**Net cost to INHS (€m)**	**QALY gains ('000 s)**	Incremental net benefit versus do-nothing (€m)*
A	Registration	FAST 3 & AUDIT 8	254	299	-45	57	1378
B	Registration	FAST 3	259	297	-39	57	1381
C	Registration	AUDIT 8	284	321	-38	62	1520
D	Registration	AUDIT 8/6	316	321	-5	62	1555
E	Registration	AUDIT-C 5/4 & AUDIT 8	356	338	17	67	1680
Next registration (baseline)	Registration	AUDIT-C 5/4	411	370	41	75	1921
F	Consultation	FAST 3 & AUDIT 8	419	503	-84	111	2684
G	Consultation	FAST 3	422	505	-84	111	2694
H	Consultation	AUDIT 8	470	500	-30	121	3000
I	Consultation	AUDIT 8/6	529	544	-15	122	3030
J	Consultation	AUDIT-C 5/4 & AUDIT 8	595	519	76	127	3258
Next consultation (baseline)	Consultation	AUDIT-C 5/4	687	605	82	139	3562

*Assuming a willingness-to-pay of €25000/QALY.

## Discussion

This adaptation of SAPM provides the first cost-effectiveness analysis of screening and brief intervention programmes in Italy, examining two implementation options: screening at the next registration with a new GP or screening at the next GP consultation. The outcome measures observed were the costs of screening, the reduction in costs to the INHS as a result of reduced morbidity and mortality and the improvement in health outcomes measured in QALYs, in line with standard practice in Italian cost-effectiveness analyses [17]. The resulting incremental cost-effectiveness ratios for all scenarios suggest that either of the modelled SBI programmes would be highly cost-effective when compared with a policy of no SBI, under current Italian guidelines [43], with a policy of SBI at next consultation using the current AUDIT-C 5/4 screening tool bringing the greatest net benefit of all modelled options (at a willingness-to-pay threshold of €25000/QALY).

The results of this study are broadly comparable with those of Purshouse et al. [11] who modelled similar programmes in England. Both studies show programmes of screening and brief interventions to be cost-effective, or even cost-saving, in a primary health care context. They show screening at next GP registration to have lower implementation costs than screening at next GP consultation, as a result of screening a smaller proportion of the population, but with reduced savings to the health service in the longer run and smaller gains in health-related quality of life. For a programme of screening at next registration, delivery costs are greater in Italy as a result of the substantially larger proportion of the population captured (63% screened, with 32% receiving a brief intervention versus 39% and 13% respectively in England). As a result hospital costs and QALY gains are also estimated to be greater, although the policy is estimated to be largely cost-neutral rather than cost-saving. The results of this analysis are also similar to those of Solberg et al. [44] in the USA who estimated an ICER of $1755 (equivalent to €1721 in 2008 prices) and Tariq et al. [45] in the Netherlands who estimated an ICER of €5400, although these studies both use substantially different methodologies.

The principal challenges to this analysis were those presented by the availability of Italian data with which to adapt the existing model. Whilst every effort was made to obtain suitable data specific to the Italian context this was not always possible, and a number of assumptions had to be made regarding the similarities of the English and Italian health care systems. Another significant limitation is the lack of available evidence on the ways in which different population subgroups respond to brief interventions, both in terms of the immediate impact on consumption and the duration of effect. Whilst several studies have attempted to investigate differential effectiveness by age and gender, a recent review of reviews identified that this evidence is still inconclusive [46]. There is some evidence that SBIs are less effective amongst young adults and older drinkers; however a recent meta-analysis estimated mean consumption reductions of 11.3% and 10.6% respectively for these populations [47], considerably above the pessimistic assumption of 5.9% tested in this study. It should also be noted that no effect is assumed for patients who have been screened, but receive no brief intervention, although a number of studies report consumption reductions in control groups under these circumstances. Whilst this may be explained by regression to the mean or an observer (or ‘Hawthorne’) effect, it may also be that screening alone has some impact as a spur to reduce consumption [46]. Further research into heterogeneity of response must be considered a high priority for decision-makers seeking to identify the populations who would benefit most from SBIs. Such studies may also wish to consider the probable impact that alcohol consumption, socioeconomic status, ethnicity and other factors may have on GP registration, consultation and screening rates.

When considering the results presented in this study, thought should be given to the relationship of both the ‘do-nothing’ comparator scenario and the modelled programme to the realities of primary care. This analysis assumes that in the absence of an SBI programme, no alternative alcohol-related interventions or advice are offered and that population consumption remains unchanged (after adjusting for changes in the age-sex distribution). Similarly the modelled programme assumes 100% of GPs participate, all eligible patients are screened at the first opportunity and no patients are screened more than once. At present the delivery in primary care in Italy of either formal SBIs or informal advice aimed at reducing patients’ drinking is likely to be infrequent and this is therefore unlikely to affect the conclusions of this study. The question of cost-effectiveness for partial uptake rates (i.e. where not every GP participates, or not every eligible patient is screened) is an important one for policy makers. The ODHIN project incorporates a major pan-European trial to investigate how different strategies (such as financial incentives or educational programmes) can improve these uptake rates [48] and we plan to utilise the results of this trial in the future to investigate the cost-effectiveness of such strategies as well as partial uptake rates. Finally, whilst the assumption that no patient is screened more than once may not hold true in practice, the published evidence on the effect of repeated brief interventions on alcohol consumption is limited and it is possible that the benefits of such repeat interventions may outweigh the additional costs of their delivery.

In the model we assume, in the absence of any alternative evidence, linear relationship between certain variables in the model. These assumptions fall into three main areas: the rebound of alcohol consumption following an SBI to pre-intervention levels; the time lags between changes in alcohol consumption and changes in risk of chronic alcohol-related harms; and the dose–response relationships between alcohol consumption and relative risk of acute alcohol-related harms. It is possible that the true relationships between these variables may be non-linear, in such a way that the non-linear reduction in effect over time post-SBI could lead a model with linear assumptions to over-estimate the benefits of the SBI programme. To mitigate this possibility, we have run a highly conservative linear sensitivity analysis in which the effect of intervention lasts only 3 years, and demonstrated that this change does not affect the overall conclusions of the study. The baseline time lag assumption is already conservative with respect to the limited available evidence, which suggests that the majority of the long-term health benefits are experienced in the first few years following a change in consumption [49]. The baseline dose–response relationships for acute conditions are also conservative from the perspective of effects on episodic heavy drinkers (who are more likely to be given a brief intervention) since candidate alternative risk functions for these conditions (e.g. quadratic or exponential forms) would have steeper gradients at higher levels of consumption, leading to greater falls in risk and therefore greater reductions in harm than in the linear case, for any given reduction in consumption.

## Conclusions

This study is the first to evaluate the cost-effectiveness of a programme of screening and brief interventions in primary care in Italy. In common with other studies internationally the results demonstrate that such programmes are highly likely to be cost-effective, even under the most pessimistic assumptions. In the present financial climate these results provide a strong recommendation for the regional Italian bodies to increase their use of SBIs as an effective tool for improving public health and reducing the burden on the INHS. However, policy makers should be mindful of the differing cost-implications of alternative programmes of implementation. Whilst screening at next GP consultation brings the greatest health benefits and affects the largest number of people, it also carries a heavily front-loaded resource profile, whereas implementing a programme of screening at next GP registration offers a much more even spread of resourcing over the duration of the programme. These differences may have a major effect on the acceptability of different SBI programme options to policy makers attempting to balance limited health care budgets.

## Abbreviations

AAF: Alcohol-attributable fraction; DHD: Dutch Hospital Data Foundation; FVG: Friuli-Venezia-Giulia; ICER: Incremental cost-effectiveness ratio; INHS: Italian National Health Service; ISTAT: Italian national institute for statistics; ODHIN: Optimising delivery of healthcare interventions; QALY: Quality-Adjusted Life Year; SBI: Screening and brief interventions; WHO: World Health Organisation.

## Competing interests

The authors declare that they have no competing interests.

## Authors' contributions

CA performed the modelling and drafted the article. ES and SG provided data and expertise on population baseline characteristics. AT and FF provided data and expertise on hospital admissions and costs. PS provided data and expertise on primary care. ES, SG, AT, FF and PS provided expertise on the Italian context and healthcare system. RP and AB provided modelling expertise and guidance. All authors read and approved the final manuscript.

## Pre-publication history

The pre-publication history for this paper can be accessed here:

http://www.biomedcentral.com/1471-2296/15/26/prepub

## Supplementary Material

Additional file 1 This file gives further details of the modelling methodology and model inputs not included in the main text. Click here for file
