# Supplementary material for: Cost-effectiveness of a programme of screening and brief interventions for alcohol in primary care in Italy
Source: BMC Fam Pract. 2014 Feb 6;15:26. doi: 10.1186/1471-2296-15-26 (PMC3936801; doi:10.1186/1471-2296-15-26)
Supplement: Additional file 1 — This file gives further details of the modelling methodology and model inputs not included in the main text. [file 1471-2296-15-26-S1.docx]

**Cost-Effectiveness of a Programme of Screening and Brief Interventions in Primary Care In Italy - Technical Appendix**

Colin Angus, Emanuele Scafato, Silvia Ghirini, Aleksandra Torbica, Francesca Ferre, Pierluigi Struzzo, Robin Purshouse, Alan Brennan

# Section 1 - Arrival Profiles

The model requires data on the proportion of people in each age-gender subgroup who will be captured by each modelled screening programme, in each year following implementation. Whilst this data was available for Italy registration with a new GP, the equivalent data was not available for GP consultations. Data was identified on the average number of consultations per annum in Italy [1] and England [2] by age and gender subgroups. Table 1 shows the Italy: England consultation ratio for each subgroup

#### Table 1 - Ratio of GP consultations Italy:UK

| **Italy/UK** | **16-17** | **18-24** | **25-34** | **35-44** | **45-54** | **55-64** | **65-74** | **75+** |
| --- | --- | --- | --- | --- | --- | --- | --- | --- |
| **Male** | 0.99 | 0.99 | 0.99 | 0.99 | 1.44 | 1.44 | 1.77 | 1.40 |
| **Female** | 0.74 | 0.74 | 0.74 | 0.74 | 1.21 | 1.21 | 1.56 | 1.16 |

As the consultation frequency amongst men aged 16-44 was almost identical in both countries, the proportion of this population presenting to their GP in each year of a programme was also assumed to be the same. For all other age-gender subgroups it was assumed that each individual has an underlying mean frequency of GP attendance per year, and that these frequencies are normally distributed, truncated below at 0 (as no patient can have a negative number of consultations). For each age-gender subgroup, 1000 individuals were simulated from this distribution, with mean equal to the observed number of visits for the UK, using the NtRand [3] add-in for Excel . For each individual it was assumed that their actual number of GP visits in a given year was Poisson distributed about their underlying mean and random draws were made from this distribution to estimate their consultation frequency in each of the 10 years of the SBI programme. This allowed an implied arrival profile to be generated from the simulated individuals, which was compared to the observed profile for the age-gender subgroup taken from the English model [4]. The standard deviation of the truncated normal distribution was then varied in order to identify the value which provided the closest match between the implied and observed profiles. Model fit was compared using root mean squared error between the observed and estimated arrival profiles for each subgroup.

Having identified the standard deviation which gave the best fit, this was assumed to be the same between the UK and Italy, and the simulation was repeated for Italy using the observed number of visits reported in [1], to generate an estimated arrival profile for that age-gender subgroup. This exercise was repeated to construct a complete profile for the entire population. In order to test the validity of this truncated normal-Poisson specification a range of alternative distributional assumptions were tested, including Poisson-Poisson, negative binomial, exponential, lognormal and lognormal-Poisson, however these did not give a satisfactory fit to the observed English arrival profile data.

The final arrival profile for Italy is given in Table 2, together with the English profile for comparison. Owing to our uncertainty surrounding the underlying assumptions of this methodology, we ran the final model using both the estimated Italian, and the observed English arrival profiles. The choice of profile made relatively little difference to the cost-effectiveness of the SBI programme, with an average difference in ICERs of less than 8% between the two across all modelled scenarios.

# Section 2 - Alcohol-Attributable Fractions

The Alcohol-Attributable Fraction (AAF) is a measure of the proportion of a disease which is attributable to alcohol. An Italian study in 2009 calculated AAFs for a range of conditions, including motor vehicle accidents (37.1% for men, 18.1% for women), fall injuries (20.6% for men, 6.9% for women), drowning (36.4% for men, 33.5% for women), accidental poisoning by exposure to noxious substances (23.3% for men, 17.7% for women), other unintentional injuries (32.7% for men, 26.1% for women), intentional self-harm (including suicide) (15.2% for men, 11.3% for women) and assault (including homicide) (35.8% for both men and women)[5]. For fire injuries and other intentional injuries Italian AAFs were unavailable, so we used the English AAFs of Jones et al.[6] (38% for men aged 16-44, 32% for men aged 45+. 31% for women aged 16-44 and 26% for women aged 45+, for both health conditions). Section 3 - Risk Functions

The SAPM requires 2 risk functions relating Relative Risk (RR) of harm (one for mortality and one for morbidity) to level of alcohol consumption for each health condition included in the model. The 42 health conditions are divided into 4 categories: wholly-attributable chronic, wholly-attributable acute, partially-attributable chronic and partially attributable acute. Risk functions for each category are obtained differently, so we will describe each in turn:

## Wholly-Attributable Chronic Conditions

These conditions are modelled as a function of mean alcohol consumption. A lower threshold of 24g/week for men and 16g/week for women is assumed, based on Italian safe drinking guidelines [7], below which the risk of harm does not increase with consumption. Due to a lack of published evidence on the shape of the relationship between mean consumption and relative risk of harm, a linear form for the risk function is assumed above this threshold. The slope of the risk functions for each age-gender subgroup is calibrated using the observed consumption distribution and number of mortalities/morbidities for that subgroup.

## Wholly-Attributable Acute Conditions

These conditions are modelled as a function of the number of times in the year the AVQ respondent reported drinking more than 6 glasses (72 grams) of alcohol in a single occasion. As consumption at this level is strongly indicative of intoxication, the risk of harm is assumed to increase linearly with every reported occasion above 0. As with wholly-attributable chronic conditions the slope of the risk functions is calibrated using the observed distribution of drinking more than 6 glasses on an occasion and number of mortalities/morbidities for that subgroup.

## Partially-Attributable Chronic Conditions

Risk functions relating mean alcohol consumption and risk of contracting or dying from a partially-attributable chronic condition are available from a wide range of published studies. Following a recent review by members of the Sheffield Alcohol Research Group a number of updated risk functions were identified. For all other conditions the risk functions presented in Brennan et al. (2008) were retained. A full list of the risk functions used for the 19 partially-attributable chronic conditions can be found in Table 3.

## Partially-Attributable Acute Conditions

As with wholly-attributable chronic conditions, the risk functions for partially-attributable acute conditions are assumed to be a linear function of the number of occasions per year where the respondent drank more than 6 glasses of alcohol, with risk increasing for any nonzero response. They are also calibrated to the observed acute consumption patterns and number of mortalities/morbidities for each age-gender subgroup. However, these conditions are not 100% alcohol-attributable and therefore the AAFs described in Section 2.2.3.5 must be used in conjunction with equation 1 below, where ${RR}_{i}$ is the relative risk of harm at consumption state i, $p_{i}$ is the proportion of the population exposed to alcohol at consumption state i and n is the number of consumption states, to calibrate the risk functions. For a full description of this calibration method please see pages 34-39 of [4].

### Equation 1 – Relationship Between AAF and Relative Risk

$AAF=\frac{\sum_{i=1}^{n} p_{i}({RR}_{i}-1)}{1+\sum_{i=1}^{n} p_{i}({RR}_{i}-1)}$

The only exception to the methods outlined above is the risk function for motor vehicle accidents. Whilst this is categorised as a partially-attributable acute condition, there is evidence to suggest that the RR of being injured or killed in a motor vehicle accident is increased for any level of alcohol consumption, rather than just at intoxicating levels [9]. In order to capture this increased risk associated with moderate levels of consumption, motor vehicle accidents are modelled as a function of mean alcohol consumption rather than the frequency of drinking more than 6 glasses of alcohol.

# Section 4 - Adjustment Coefficients

Data was obtained from Dutch Hospital Data Foundation (DHD), consisting of all reported hospital attendances for 2010 in the Netherlands which included, amongst the diagnoses, one of the 42 alcohol-attributable conditions included in the model. The data covers 888,838 hospital admissions, with each one including the hospital ID no., a unique patient ID no., the patient’s age and sex, the type of attendance (clinical or outpatient), the primary diagnosis and up to 17 subsidiary diagnoses. As the patient ID is assigned by the hospital there is no way of identifying patients who have attended multiple hospitals in the same year, however following discussions with ODHIN collaborators Miranda Laurant and Myrna Keurhorst in the Netherlands this was considered to be a sufficiently rare occurrence not to bias the results. 123,768 admissions (13.9%) are missing a patient ID no (i.e. repeat admissions cannot be identified) and these patients are excluded from the analysis, leaving a sample size of 765,070 admissions.

DHD collect data using ICD-9 codes, so these diagnoses were converted to ICD-10 codes using the equivalence given in Table 4 below. The ICD-9 code 577.1 doesn’t distinguish between alcohol-induced chronic pancreatitis and non-alcohol-induced chronic pancreatitis, so the UK ratio of admissions for the two harms was used to apportion diagnoses coded 577.1 between the two (100% of male and 74% of female admissions were recorded as alcohol-induced).

The aim of this analysis was to estimate the average number of hospital admissions per year for somebody with each of the 42 health conditions included in the model. As the SAPM does not explicitly model co-morbidity it was therefore necessary to select a single health condition for each individual and retain only those admissions for that individual which related to that condition. This was done following the methodology of Jones et al. (2008) who performed similar calculations using English data. From this reduced dataset containing a single diagnosis and number of hospital admissions for each individual, the mean number of admissions for each condition could be calculated, which is exactly the adjustment coefficient used in the model.

# References

1. Brignoli O, Cricelli C, Cricelli I, Innocenti F, Lapi F, Mazzaglia G, Niccolai C, Pasqua A, Pecchioli S, Sessa E, Simonetti M, Sini G: *VI Report Health Search*. Firenze; 2010:53–54.

2. Hippisley-Cox J, Vinogradova Y: *Trends in Consultation Rates in General Practice 1995/1996 to 2008/2009: Analysis of the QResearch Database*. Nottingham; 2009:1–24.

3. **www.ntrand.com** [www.ntrand.com]

4. Purshouse R, Brennan A, Latimer N, Meng Y, Rafia R, Jackson R, Meier P: *Modelling to Assess the Effectiveness and Cost-Effectiveness of Public Health Related Strategies and Interventions to Reduce Alcohol Attributable Harm in England Using the Sheffield Alcohol Policy Model Version 2.0*. Sheffield; 2009.

5. Scafato E, Ghirini S, Galluzzo L, Farchi G: *Epidemiologia e Monitoraggio Alcol-Correlato in Italia. Valutazione dell’Osservatorio Nazionale Alcol-CNESPS Sull'impatto Dell'uso e Abuso Di Alcol Ai Fini Dell'implementazione Dell Attivita Del Piano Nazionale Alcol e Salute*. Roma; 2009.

6. Jones L, Bellis M, Dedman D, Sumnall H, Tocque K: *Alcohol-Attributable Fractions for England Alcohol-Attributable Mortality and Hospital Admissions*. Liverpool: North West Public Health Observatory, Centre for Public Health Research Directorate, Liverpool John Morres University, Liverpool; 2008.

7. Istituto Nazionale di Ricerca per gli Alimenti e la Nutrizione (INRAN): *Linee Guida Per Una Sana Alimentazione Italiana: Revisione 2003*. 2003.

8. Brennan A, Purshouse R, Taylor K, Rafia R, Booth A, O’Reilly D, Stockwell T, Sutton A, Wilkinson A, Wong R: *Independent Review of the Effects of Alcohol Pricing and Promotion. Part B: Modelling the Potential Impact of Pricing and Promotion Policies for Alcohol in England. Results from the Sheffield Alcohol Policy Model Version 2008 (1.1)*. Sheffield; 2008.

9. Taylor B, Irving H, Kanteres F, Room R, Borges G, Cherpitel C, Greenfield T, Rehm J: **The more you drink, the harder you fall: a systematic review and meta-analysis of how acute alcohol consumption and injury or collision risk increase together**. *Drug Alcohol Depend* 2010, **110**:108–116.

10. Tramacere I, Negri E, Bagnardi V, Garavello W, Rota M, Scotti L, Islami F, Corrao G, Boffetta P, La Vecchia C: **A meta-analysis of alcohol drinking and oral and pharyngeal cancers. Part 1: overall results and dose-risk relation.** *Oral Oncol* 2010, **46**:497–503.

11. Corrao G, Bagnardi V, Zambon A, La Vecchia C: **A meta-analysis of alcohol consumption and the risk of 15 diseases.** *Prev Med (Baltim)* 2004, **38**:613–9.

12. Islami F, Tramacere I, Rota M, Bagnardi V, Fedirko V, Scotti L, Garavello W, Jenab M, Corrao G, Straif K, Negri E, Boffetta P, La Vecchia C: **Alcohol drinking and laryngeal cancer: overall and dose-risk relation--a systematic review and meta-analysis.** *Oral Oncol* 2010, **46**:802–10.

13. Key J, Hodgson S, Omar RZ, Jensen TK, Thompson SG, Boobis AR, Davies DS, Elliott P: **Meta-analysis of studies of alcohol and breast cancer with consideration of the methodological issues.** *Cancer causes Control CCC* 2006, **17**:759–70.

14. Gutjahr E, Gmel G, Rehm J: **Relation between average alcohol consumption and disease: an overview.** *Eur Addict Res* 2001, **7**:117–27.

15. Samokhvalov A V, Irving H, Mohapatra S, Rehm J: **Alcohol consumption, unprovoked seizures, and epilepsy: a systematic review and meta-analysis.** *Epilepsia* 2010, **51**:1177–84.

16. Kodama S, Saito K, Tanaka S, Horikawa C, Saito A, Heianza Y, Anasako Y, Nishigaki Y, Yachi Y, Iida KT, Ohashi Y, Yamada N, Sone H: **Alcohol consumption and risk of atrial fibrillation: a meta-analysis.** *J Am Coll Cardiol* 2011, **57**:427–36.

|  | | Years from inception of SBI programme | | | | | | | | | | |
| --- | --- | --- | --- | --- | --- | --- | --- | --- | --- | --- | --- | --- |
| Italy: | | 0 | 1 | 2 | 3 | 4 | 5 | 6 | 7 | 8 | 9 | Never |
| Male | 16-44 | 64.62% | 13.02% | 5.84% | 3.13% | 1.89% | 1.23% | 0.85% | 0.61% | 0.45% | 0.33% | 8.02% |
| Male | 45-64 | 89.73% | 5.33% | 1.75% | 0.92% | 0.55% | 0.39% | 0.22% | 0.22% | 0.16% | 0.07% | 0.66% |
| Male | 65-74 | 98.38% | 0.75% | 0.24% | 0.08% | 0.12% | 0.11% | 0.04% | 0.02% | 0.02% | 0.03% | 0.21% |
| Male | 75+ | 98.64% | 0.63% | 0.26% | 0.08% | 0.08% | 0.10% | 0.06% | 0.02% | 0.03% | 0.02% | 0.08% |
| Female | 16-44 | 83.62% | 9.10% | 2.72% | 1.34% | 0.73% | 0.54% | 0.33% | 0.31% | 0.21% | 0.10% | 1.00% |
| Female | 45-64 | 93.34% | 3.31% | 1.22% | 0.59% | 0.39% | 0.17% | 0.19% | 0.11% | 0.11% | 0.07% | 0.50% |
| Female | 65-74 | 99.09% | 0.44% | 0.18% | 0.08% | 0.01% | 0.01% | 0.03% | 0.04% | 0.03% | 0.01% | 0.08% |
| Female | 75+ | 97.05% | 1.35% | 0.61% | 0.25% | 0.16% | 0.05% | 0.05% | 0.05% | 0.04% | 0.04% | 0.35% |
|  | | | | | | | | | | | | |
| England: | | 0 | 1 | 2 | 3 | 4 | 5 | 6 | 7 | 8 | 9 | Never |
| Male | 16-44 | 64.62% | 13.02% | 5.84% | 3.13% | 1.89% | 1.23% | 0.85% | 0.61% | 0.45% | 0.33% | 8.02% |
| Male | 45-64 | 73.43% | 9.68% | 4.20% | 2.21% | 1.32% | 0.86% | 0.59% | 0.42% | 0.31% | 0.23% | 6.73% |
| Male | 65-74 | 89.54% | 4.23% | 1.65% | 0.81% | 0.47% | 0.30% | 0.20% | 0.14% | 0.11% | 0.08% | 2.47% |
| Male | 75+ | 94.94% | 2.06% | 0.79% | 0.39% | 0.22% | 0.14% | 0.10% | 0.07% | 0.05% | 0.04% | 1.19% |
| Female | 16-44 | 85.49% | 7.09% | 2.71% | 1.28% | 0.70% | 0.43% | 0.28% | 0.20% | 0.14% | 0.10% | 1.58% |
| Female | 45-64 | 89.37% | 5.79% | 2.07% | 0.74% | 0.30% | 0.18% | 0.11% | 0.07% | 0.05% | 0.04% | 1.28% |
| Female | 65-74 | 94.21% | 3.13% | 1.09% | 0.48% | 0.25% | 0.15% | 0.10% | 0.07% | 0.05% | 0.03% | 0.45% |
| Female | 75+ | 95.06% | 2.24% | 0.86% | 0.42% | 0.23% | 0.15% | 0.10% | 0.07% | 0.05% | 0.04% | 0.79% |

### Table 2 - Proportion of population screened in each year following the inception of a programme of SBI at next GP consultation. Estimated Italian profile and observed English profile for comparison.

| **Condition** | **Risk function** | | **Source** |
| --- | --- | --- | --- |
|  |  |  |  |
| Malignant neoplasm of lip, oral cavity and pharynx | $\ln\left( RR \right)=0.02572x-0.00006x^{2}$ | | Tramacere et al.[10] |
| Malignant neoplasm of oesophagus | $\ln\left( RR \right)=0.0133x$ | | Corrao et al.[11] |
| Malignant neoplasm of colon and rectum | $\ln\left( RR \right)=-0.00000000000000002+0.002x$ | |  |
| Malignant neoplasm of liver and intrahepatic bile ducts | $\ln\left( RR \right)=0.00743x-0.00001x^{2}$ | |  |
| Malignant neoplasm of larynx | $\ln\left( RR \right)=0.01625x-0.00003x^{2}$ | | Islami et al.[12] |
| Malignant neoplasm of breast | $RR=1+0.001x$ | | Key et al.[13] |
| Diabetes mellitus (type II) | Male | $RR=1.0464-0.0139x+0.0001x^{2}$ | Gutjahr et al.[14] |
|  | Female | $RR=1.0261-0.0103x+0.0002x^{2}$ |  |
| Epilepsy and status epilepticus | $\ln\left( RR \right)=0.006143+0.012286x$ | | Samokhvalov et al.[15] |
| Hypertensive diseases | $\ln\left( RR \right)=-0.0000000000000002+0.0146x$ | | Corrao et al.[11] |
| Ischaemic heart disease | Male | $\ln\left( RR \right)=-0.1065\sqrt{x}+0.01454x$ |  |
|  | Female | $\ln\left( RR \right)=-0.1065\sqrt{x}+0.0207x$ |  |
| Cardiac arrhythmias | $RR=1+0.008x$ | | Kodama et al.[16] |
| Haemorrhagic stroke | $\ln\left( RR \right)=-0.057+0.0058x+0.0002x^{2}-0.000001x^{3}$ | | Corrao et al.[11] |
| Ischaemic stroke | $\ln\left( RR \right)=-0.0432+0.0095x+0.0003x^{2}-0.0000006x^{3}$ | |  |
| Oesophageal varices | $\ln\left( RR \right)=0.0668+0.0444x-0.0001x^{2}$ | |  |
| Unspecified liver disease | $\ln\left( RR \right)=0.0668+0.0444x-0.0001x^{2}$ | |  |
| Cholelithiasis | Male | $RR=0.9884-0.0075x+0.00002x^{2}$ | Gutjahr et al.[14] |
|  | Female | $RR=0.9803-0.0134x+0.00009x^{2}$ |  |
| Acute and chronic pancreatitis | $\ln\left( RR \right)=0.012x$ | | Corrao et al.[11] |
| Psoriasis | Male | $RR=1.0945+0.014x-0.00001x^{2}$ | Gutjahr et al.[14] |
|  | Female | $RR=1.1295+0.0226x-0.00009x^{2}$ |  |
| Spontaneous abortion | $RR=0.953+0.0371x-0.0004x^{2}$ | |  |

### Table 3 - Risk functions for partially-attributable chronic conditions.

| **Health Condition** | **ICD-10 Code(s)** | **ICD-9 Code (s)** |
| --- | --- | --- |
| **Wholly Alcohol-Attributable Chronic Conditions** | | |
| Alcohol-induced pseudo-Cushing's syndrome | E24.4 | 255 |
| Degeneration | G31.2 | 331.7 |
| Alcoholic polyneuropathy | G62.1 | 357.5 |
| Alcoholic myopathy | G72.1 | 359.4 |
| Alcoholic cardiomyopathy | I42.6 | 425.5 |
| Alcoholic gastritis | K29.2 | 535.3 |
| Alcoholic liver disease | K70 | 571.0-571.3 |
| Chronic pancreatitis | K86.0 | 577.1 |
| **Wholly Alcohol-Attributable Acute Conditions** | | |
| Excessive blood level of alcohol | R78.0 | 790.3 |
| Mental and behavioural disorders due to use of alcohol | F10 | 291, 303 |
| Ethanol poisoning | T51.0 | 980 |
| Methanol poisoning | T51.1 | 980.1 |
| Toxic effect of alcohol, unspecified | T51.9 | 980.9 |
| Accidental poisoning by exposure to alcohol | X45 | E860 |
| **Partially Alcohol-Attributable Chronic Conditions** | | |
| Malignant neoplasm of lip, oral cavity and pharynx | C00-C14 | 141, 143-146, 148, 149 |
| Malignant neoplasm of oesophagus | C15 | 150 |
| Malignant neoplasm of colon and rectum | C18-21 | 153-154 |
| Malignant neoplasm of liver and intrahepatic bile ducts | C22 | 155 |
| Malignant neoplasm of larynx | C32 | 161 |
| Malignant neoplasm of breast | C50 | 174 |
| Diabetes mellitus (typeII) | E10-E14 | 250 |
| Epilepsy and status epilepticus | G40-G41 | 345 |
| Hypertensive diseases | I10-I15 | 401-405 |
| Ischaemic heart disease | I20-I25 | 410-414 |
| Cardiac arrhythmias | I47-I49 | 427 |
| Haemorrhagic stroke | I60-I62 | 430-432 |
| Ischaemic stroke | I63 | 433-437 |
| Oesophageal varices | I85 | 456.0-456.2 |
| Unspecified liver disease | K74 | 571.5-571.9 |
| Cholelithiasis | K80 | 574 |
| Acute and chronic pancreatitis | K85, K86.1 | 577.0-577.1 |
| Psoriasis | L40 excl. L40.5 | 696.0-696.2 |
| Spontaneous abortion | O03 | 634 |
| **Partially Alcohol-Attributable Acute Conditions** | | |
| Motor Vehicle Accidents | V0-V04, V06, V09-V80, V87, V89, V99 | E810-819 |
| Fall injuries | W00-W19 | E880-E888, E848 |
| Drowning | W65-W74 | E910 |
| Fire injuries | X00-X09 | E890-E899 |
| Accidental poisoning by exposure to noxious substances | X40-X49 | E850-E858, E861-E869 |
| Other Unintentional Injuries | V05, V07, V08, V81-V86, V88, V90-V98, W20-W64, W75-W99, X10-X39, X50-X59, Y40, Y86, Y88, Y89 | E800-E849, E870-E879, E900-E909, E911-E929 |
| Intentional self-harm | X60-X84, Y87.0 | E950-E959 |
| Assault | X85-Y09, Y87.1 | E960-E969 |
| Other Intentional Injuries | Y35 | E970-E978 |

### Table 4 - ICD 9 - 10 mapping
